# Diagnosis of Mesothelioma Using Image Segmentation and Class-Based Deep Feature Transformations

**DOI:** 10.3390/diagnostics15182381

**Published:** 2025-09-18

**Authors:** Siyami Aydın, Mehmet Ağar, Muharrem Çakmak, Mesut Toğaçar

**Affiliations:** 1 Department of Thoracic Surgery, Faculty of Medicine, Fırat University, Elazığ 23119, Türkiye; md.mehmetagar@gmail.com (M.A.); g.c.dr.ckmk@gmail.com (M.Ç.); 2 Department of Management Information Systems, Faculty of Economics and Administrative Sciences, Fırat University, Elazığ 23119, Türkiye; mtogacar@firat.edu.tr

**Keywords:** mesothelioma disease, mesothelioma detection, transformer-based segmentation, class-based feature, deep generative, discriminative score

## Abstract

**Background/Objectives**: Mesothelioma is a rare and aggressive form of cancer that primarily affects the lining of the lungs, abdomen, or heart. It typically arises from exposure to asbestos and is often diagnosed at advanced stages. Limited datasets and complex tissue structures contribute to delays in diagnosis. This study aims to develop a novel hybrid model to improve the accuracy and timeliness of mesothelioma diagnosis. **Methods**: The proposed approach integrates automatic image segmentation, transformer-based model training, class-based feature extraction, and image transformation techniques. Initially, CT images were processed using the segment anything model (SAM) for region-focused segmentation. These segmented images were then used to train transformer models (CaiT and PVT) to extract class/type-specific features. Each class-based feature set was transformed into an image using Decoder, GAN, and NeRV techniques. Discriminative score and class centroid analysis were then applied to select the most informative image representation for each input. Finally, classification was performed using a residual-based support vector machine (SVM). **Results**: The proposed hybrid method achieved a classification accuracy of 99.80% in diagnosing mesothelioma, demonstrating its effectiveness in handling limited data and complex tissue characteristics. **Conclusions**: The results indicate that the proposed model offers a highly accurate and efficient approach to mesothelioma diagnosis. By leveraging advanced segmentation, feature extraction, and representation techniques, it effectively addresses the major challenges associated with early and precise detection of mesothelioma.

## 1. Introduction

Malignant mesothelioma (MM) is a rare and aggressive tumor that originates in the mesothelial cells that line the pleura, peritoneum, pericardium, or tunica vaginalis. Malignant pleural mesothelioma (MPM) constitutes nearly 80% of all cases and is primarily associated with prolonged asbestos exposure [1]. Despite global regulations limiting asbestos use, the disease burden persists due to its long latency period, which is often 20 to 50 years [2].

MM presents insidiously with symptoms such as progressive dyspnea, pleuritic chest pain, chronic cough, fatigue, and unintentional weight loss. These nonspecific symptoms often result in delayed diagnoses; more than 60% of patients are diagnosed at advanced stages. Physical examination findings may include decreased breath sounds and dullness to percussion [3]. Paraneoplastic manifestations, such as thrombocytosis or digital clubbing, may also be observed. Diagnosis typically requires a combination of imaging, histopathology, and molecular testing. Chest CT is the initial imaging modality and often reveals unilateral pleural thickening or effusion. PET-CT can aid in staging. However, tissue biopsy remains the gold standard for diagnosis. Immunohistochemistry is essential for distinguishing MM from other malignancies, particularly metastatic adenocarcinoma [4]. Recent advances in artificial intelligence (AI)-assisted imaging analysis show promise in improving diagnostic accuracy by identifying radiographic patterns predictive of MM. This could allow for earlier and more reliable detection [5].

MPM is pathologically classified into three subtypes: epithelioid, sarcomatoid, and biphasic. The epithelioid subtype has a better prognosis, while the sarcomatoid and biphasic variants are more resistant to therapy. Molecular profiling is playing an increasingly important role in guiding treatment, particularly in identifying candidates for targeted therapies and immunotherapies. Historically, the first-line treatment for unresectable MPM has been a combination of cisplatin and pemetrexed, which offers limited survival benefit. However, the therapeutic landscape has changed significantly in recent years. In 2020, the FDA approved the combination of the immune checkpoint inhibitors nivolumab and ipilimumab as a first-line treatment for unresectable MPM. The pivotal CheckMate 743 trial demonstrated a median overall survival of 18.1 months with immunotherapy versus 14.1 months with chemotherapy [6].

Further therapeutic advancements include using ADI-PEG20, an enzyme that depletes arginine, to treat ASS1-deficient mesothelioma. A recent randomized phase 2/3 trial (ATOMIC-Meso) reported a significant improvement in overall survival with the addition of ADI-PEG20 to pemetrexed and cisplatin [7].

Experimental studies are investigating the utility of AI in treatment planning and prognostication. Algorithms trained on multimodal data (histopathology, genomics, and imaging) may soon enable clinicians to personalize treatment regimens more effectively and predict responses to immunotherapy more accurately [8]. Malignant mesothelioma remains a devastating disease with a poor prognosis and limited treatment options. Nevertheless, recent progress in immunotherapy, targeted agents, and AI-assisted diagnostics offers hope for earlier diagnosis and improved outcomes. Continued interdisciplinary research is essential to translating these innovations into routine clinical practice. We will examine some recent studies in the literature that have implemented AI-based mesothelioma diagnosis.

Kitajima et al. [9] designed a three-dimensional deep convolutional neural network (3D-CNN) based on PET/CT data and successfully distinguished MPM diagnosis from benign types with an overall accuracy of 77.3%. Gill et al. [10] used a limited number of biological and radiological parameter data (pleural effusion, pH, C-reactive protein, etc.) for mesothelioma diagnosis and employed gradient-boosted trees (GBTs), support vector machines (SVMs), and logistic regression (LR) methods. They achieved 100% overall accuracy in their study. Cheng et al. [11] analyzed biological and radiological parameter data for mesothelioma diagnosis using machine learning methods. In their study, they obtained the best results with the random forest (RF) method, achieving an overall accuracy of 86.54%. Kidd et al. [12] used CT images for mesothelioma diagnosis. They performed segmentation using the U-Net model and then classification using the CNN model they designed. They achieved an overall accuracy of 66.67% in the binary classification process. A. Choudhury [13] used clinical and biomarker data to diagnose mesothelioma. In the experimental analysis, he used various machine learning methods and obtained the best result with the AdaBoost method. With the AdaBoost method, he achieved an overall accuracy of 71.29%.

Unlike previous studies, this study proposes a hybrid model that combines new approaches for MPM diagnosis. The proposed model involves the following steps: segmentation; model training; extraction of class-based feature sets from the models; conversion of these feature sets into image features; and classification. The contributions of the proposed approach to the literature are as follows:

- Use of the segment anything model (SAM) for region-focused segmentation of CT images.
- Integration of transformer-based models for class-based feature extraction.
- New use of class-based image transformation through Decoder, GAN, and NeRV techniques.
- Application of a discriminative score-based selection to determine the most informative image representations.
- Achieving high diagnostic accuracy through residual-based SVM classification.

The remaining sections of the article are summarized as follows: materials and methods are presented in Section 2; experimental analyses are presented in Section 3; comparison and discussion of the analysis results are presented in Section 4; and conclusions and information about future studies are presented in Section 5.

## 2. Materials and Methodology

### 2.1. CT Image Dataset

The dataset is not publicly accessible and was created by specialists in the field. The images included in the dataset were obtained from Fırat University Research Hospital. The retrospective study included a total of 172 patients, comprising 86 patients diagnosed with mesothelioma based on radiological and pathological findings between 2012 and 2024, and 86 patients investigated for suspected mesothelioma but who did not receive a mesothelioma diagnosis. A total of 1008 sections were included in the study, with an average of 5.8 chest CT images obtained from each group (mesothelioma and non-mesothelioma). Of the patients included in the study, 98 were male and 74 were female. The average age was 56.8 ± 14.6 years. In the staging, stage 3 was the most common, and the most common pathological diagnosis was epithelioid-type mesothelioma.

**Figure 1:**
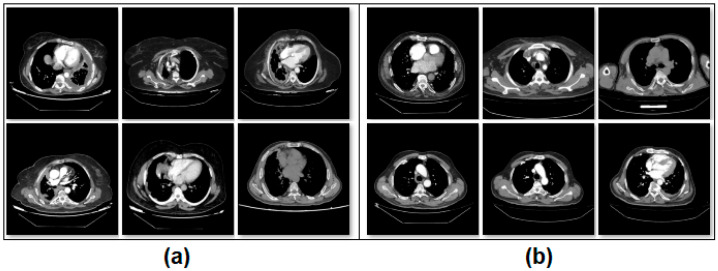
Class-based sample images of the dataset; (**a**) mesothelioma, (**b**) non-mesothelioma.

Asbestos exposure was present in 131 (76.2%) of the patients, and a history of smoking was present in 97 (56.4%) of the patients. Of the patients, 102 (59.3%) reported chest wall or chest pain, and 115 (66.9%) had weight loss. Detailed information is provided in Table 1. The dataset consists of two classes. The number of images from patients diagnosed with mesothelioma is 504. The number of patients without a mesothelioma diagnosis but with images suggestive of mesothelioma is 504. The non-mesothelioma group in the dataset includes only benign pleural diseases; other subgroups, such as metastatic pleural diseases and pleural effusion, are not included in this assessment. Benign pleural diseases include pleural plaques, infection-related conditions, inflammatory diseases, and findings related to trauma or surgery. This dataset was created to compare individuals diagnosed with mesothelioma with individuals who initially had suspected mesothelioma but were ultimately diagnosed with benign pleural disease after further investigation. The images in the dataset are equally classified and consist of a total of 1008 images. All CT slices corresponding to a single patient were kept in the same subset (either training or test), ensuring no overlap of patient data across sets. Since CT imaging inherently provides multiple slices at different depths and angles, all slices belonging to the same patient, regardless of orientation, depth, or reconstruction parameters, were grouped within the same subset. This patient-based separation ensured the independence of training and test datasets, eliminating the risk of data leakage. A sample image set is shown in Figure 1.

### 2.2. Segment Anything Model

The segment anything model (SAM) is a segmentation approach that distinguishes objects in images and processes them using a masking technique. The SAM can focus on different types of commands, such as single or multiple points or textual regions, within an image. These commands are specified before running the model. One of the SAM’s key advantages is its ability to perform meaningful segmentation on image sets, even when commands are ambiguous. This success is underpinned by the model’s prior learning of object concepts. This feature enables successful results to be achieved even on untrained test examples [14]. During SAM training, pre-trained large data files (e.g., “sam vit-h”) are used. The core modules in the model’s architectural structure perform the following tasks:

- Image Encoder: Processes the input image and extracts visual information. This information is converted into a high-dimensional and deep visual representation. The resulting representation is prepared for use in the next stage.
- Prompt Encoder: Processes commands provided by the user (e.g., a single point, multiple points, etc.). These commands determine which part of the image the model should focus on. The commands are converted into vectors that the model can understand.
- Mask Decoder: Combines the image representations and command vectors obtained from the previous two stages to create a segmentation mask for the focused area. In short, it predicts which pixel belongs to which object. The SAM can generate multiple masks probabilistically; however, the mask with the highest probability is selected during the process [15].

The reasons for choosing the SAM in this study are as follows: its ability to operate using command-based inputs (such as points, text, boxes, etc.), its capability to distinguish even previously undefined objects, its ability to differentiate between various types of objects, its ability to process an image only once to produce faster results, and its provision of different masking options to apply the most suitable/likely mask to the image.

### 2.3. Transformer Models

**Figure 2:**
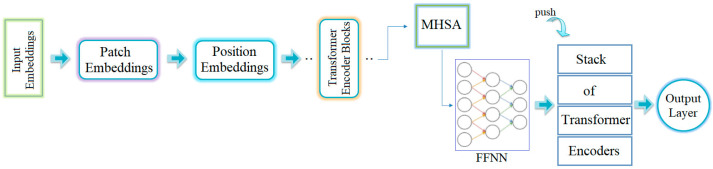
General workflow process of the transformer model [16].

Transformer models were first developed for text-based applications. However, in recent years, they have been adapted to visual-based datasets due to their successful results in image processing. At the core of transformer models lies the division of an image into small patches. The relationship between each patch and the others is modeled through multi-head self-attention (MHSA). Visual features present in the patches are extracted by the model and passed on to the next layer as a sequence. The extracted information is processed using feedforward neural networks (FFNNs) to perform feature extraction [16]. These steps are summarized in Figure 2.

The Class-Attention in Image Transformers (CaiT) model was developed to address challenges in training the ViT model more deeply. It uses a unique attention module architecture called “Class-Attention.” This module ensures that attention is focused only on class-specific features. Unlike ViT models, CaiT uses the “LayerScale” module, which normalizes input values by applying a different mathematical operation to them. CaiT achieves better results in deep architectures [17].

The Pyramid Vision Transformer (PVT-v2) is a transformer-based approach designed to perform various object detection tasks with dense segmentation. It serves as a multimodal backbone. Its layers process the input image from low to high in a pyramid structure at different resolution levels. This structure enables more efficient results with fewer parameters. The PVT-v2 architecture incorporates a “spatial-reduction attention” module that reduces computational costs and resource usage. The model has demonstrated high performance in classification and segmentation tasks [18].

The reasons for choosing two different transformer models in this study can be summarized as follows: CaiT enables more efficient training of the model thanks to its deep-layer architecture. In addition, it improves model performance by focusing directly on target object features through attention mechanisms specific to class information. Furthermore, approaches such as “LayerScale” have made the training process of the CaiT model more stable. PVT-v2, on the other hand, offers the advantage of capturing both the general structure and detailed features by processing input images at variable resolutions, unlike many other transformers. This feature reduces training costs and enables the creation of a lighter and faster model. Both models have a complementary structure thanks to their unique features in addition to their common architectural structure. Another reason for preferring multiple models in hybrid approaches is the high potential for deficiencies in one model to be compensated for by other models.

### 2.4. Deep Generative Approaches

Deep generative approaches are techniques that stand out in AI-based technologies. These methods aim to produce realistic input data using their own architectures. These approaches can process features extracted from complex, high-dimensional images to generate examples similar to the original image [19].

#### 2.4.1. DecoderMLP

These approaches, which are often used in generative architectures, focus on solving latent representations through deep processing. The result is a reconstructed image that represents the original image. During reconstruction, multi-layer perceptrons (MLPs) recreate the spatial and temporal characteristics of the input data. The decoder component decodes the information encoded in the input data and converts it into an image that represents the original. This technique can also be applied to features obtained from the final fully connected (FC) layers of deep learning models or transformer architectures. This enables the processing of more meaningful features, thereby improving the accuracy of the representative images [20]. The operation performed in the Lth layer of the Decoder-MLP technique is formulated in Equation (1). In this equation, $h^{\left(L\right)}$ represents the activation output in layer L. The other parameters in Equation (1) represent the hidden vector values $h^{\left(L-1\right)}$, the weight parameter W, the bias setting $b^{L}$, and the activation function σ. In Equation (2), z: latent representation, x: pixel vector output in an image, $f_{dec}$: MLP-based decoder function, and S: total number of layers.

(1) $$h^{\left(L\right)}=\sigma (W^{\left(L\right)}h^{\left(L-1\right)}+b^{L})$$

(2) $$x=f_{dec}\left(z\right)=h^{\left(S\right)}$$

The Decoder-MLP architecture consists of multiple fully connected layers, each followed by an activation function, designed to transform latent feature representations into image outputs. The operation of the L-th layer is formally defined in Equations (1) and (2). In Equation (1), ReLU is used for hidden layers to mitigate vanishing gradients and accelerate convergence, while Tanh is applied in the final layer to scale outputs to [−1, 1]. Each layer progressively improves the spatial and temporal qualities of the input features, enabling the reconstruction of images that accurately reflect the original data.

#### 2.4.2. GAN Generator

Generative Adversarial Networks (GANs) create new, realistic data by using parts of existing data that machines have learned. A GAN architecture consists of two main components: a generator and a discriminator. The generator takes a randomly selected noise vector and meaningfully distributes it in the target image space, thereby producing fake images similar to the target images. This process operates through a learned function, with deep learning architectures, such as CNNs and MLPs, playing an active role. The generator’s primary goal is to produce realistic images that can fool the discriminator. During GAN training, the generator and discriminator take on opposing yet complementary roles: while the generator produces fake but realistic data, the discriminator attempts to distinguish whether this data is real or fake [21,22]. Through this adversarial learning process, feature sets extracted from the FC layers of transformer or CNN models can be converted into 2D GAN images. These fake images can then serve as more efficient representations of each example in the original dataset and be transferred to the output layer.

#### 2.4.3. NeRV-like

The Neural Representations for Videos (NeRV) method has a unique architecture that processes video-based data directly with neural networks. Unlike traditional pixel-based video processing methods, NeRV processes each frame by considering the time index of the extracted frames through a continuous function. This enables the generation of new image frames. In this structure, the decoder component uses structural components, such as FC layers, MLPs, and convolutional encoder–decoders, to generate output similar to GAN architectures. A key feature distinguishing NeRV from similar approaches is its ability to detect temporal continuity in frames and convert it into relationships between patterns using vector representation spaces. In NeRV, rather than storing each frame separately, the architecture encodes each frame in a reproducible manner based on its time index. This approach ensures preservation of image quality with less resource usage. NeRV can be effectively used in various video processing tasks such as compression, reconstruction, editing, and data transfer. Its architecture consists of a temporal encoder and a temporal decoder. The temporal encoder converts the information obtained from the input data into high-dimensional feature vectors. These vectors are then processed by the decoder and reproduced in a sequential structure for each frame. Another important advantage of NeRV is its ability to generate frames at specific points in time through the neural network during operation. This capability fills in missing areas of frames and prevents resolution errors [23,24]. In recent years, NeRV-like methods have been combined with new approaches and have begun to be used in areas such as generative image creation.

The NeRV-like architecture includes a temporal encoder that converts input video frames into high-dimensional vectors. These vectors are then processed by a decoder consisting of convolutional and fully connected layers, which enable the sequential reconstruction of each frame. This structure enables efficient frame generation while preserving temporal continuity, without the need to store each frame separately.

### 2.5. Discriminative Score Method

The discriminative score (DS) is a method developed in fields such as machine learning, deep learning, and pattern recognition, which are subfields of artificial intelligence, to select the most discriminating feature among image sets or features representing these images. This method consists of two basic steps. First, feature vectors are extracted from examples belonging to each class, and then the class centers (average vectors) of these vectors are calculated. In the second step, the distances between each example and its own class center and other class centers are calculated. For effective discrimination, it is expected that the distance of a sample from its own class center is minimum, while the distances from other class centers are maximum. The DS identifies the most discriminative sample by considering these differences. The Euclidean distance is generally preferred for this measurement. The sample with the lowest DS is the one closest to its own class and farthest from other classes, and is therefore considered the best representative sample [25,26]. The general formula used in calculating the DS is given in Equation (3). In Equation (3), $m_{i}$ represents the sample to be examined, $C_{poz}$ represents the center vector of the class in which $m_{i}$ is located (positive class), and $C_{neg}$ represents the center vector of the class in which $m_{i}$ is not located but is the closest class (negative class). “d” calculates the distance between the two values using the Euclidean formula.

(3) $$DS\left(m_{i}\right)=\frac{d(m_{i},C_{neg})}{d(m_{i},C_{poz})}$$

This method is particularly useful for selecting images created using generative techniques or for measuring the discriminative power between variants. This enables the selection of more efficient images, thereby contributing to the improvement of the training performance of machine learning or deep learning models.

### 2.6. Proposed Hybrid Approach

Mesothelioma is a rare, rapidly progressing type of pleural cancer. In its early stages, it can be difficult to diagnose and is often mistaken for other respiratory diseases by specialists. This makes it difficult to diagnose the disease in a timely manner. Ultimately, many cases are detected at an advanced stage, when treatment options are limited. In this context, there is an increasing need for digital solutions that enable early and reliable diagnoses while remaining applicable in clinical settings. This study proposes an advanced, AI-driven, multi-layered, hybrid model to support the mesothelioma diagnosis process. The proposed model consists of three steps.

The first step (step #1) involves processing the original dataset using the SAM approach and performing regional segmentation on the CT images. After segmentation, each region is processed to increase transparency by at least 50%, revealing the original background image. This method allows the segmented area and the tissue to which it belongs to be displayed simultaneously, enabling distinctive features to be highlighted more clearly.

In the second step (step #2), the SAM-segmented dataset is trained using transformer-based CaiT and PVT-v2 models, known as next-generation models. Under normal conditions, 384 features are extracted from the FC layer (final layer) of the CaiT model for each image; similarly, 512 features are extracted from the FC layer of the PVT-v2 model for each image. The fact that these two models produce different numbers of feature columns stems from their architectural design. However, by adding a new layer (logits) to the final layer of both models (CaiT and PVT-v2), a feature set of size [number of images × number of classes] is created. Since the dataset used in this study consists of two classes (mesothelioma and non-mesothelioma), 2 features are extracted from the logits layer for each image during model training. We refer to this process as “class-based feature extraction.” Class-based feature extraction typically produces more efficient features compared to the FC layer. In this study, the “F1” and “F2” feature columns were extracted from the logits layer for the CaiT model, while the “F3” and “F4” feature columns were extracted for the PVT-v2 model. In the CaiT model, the “F1” feature column represents the probability of being class #1, while the “F2” feature column represents the probability of being class #2. Similarly, in the PVT-v2 model, the “F3” feature column represents the probability of being class #1, while the “F4” feature column represents the probability of being class #2.

**Figure 3:**
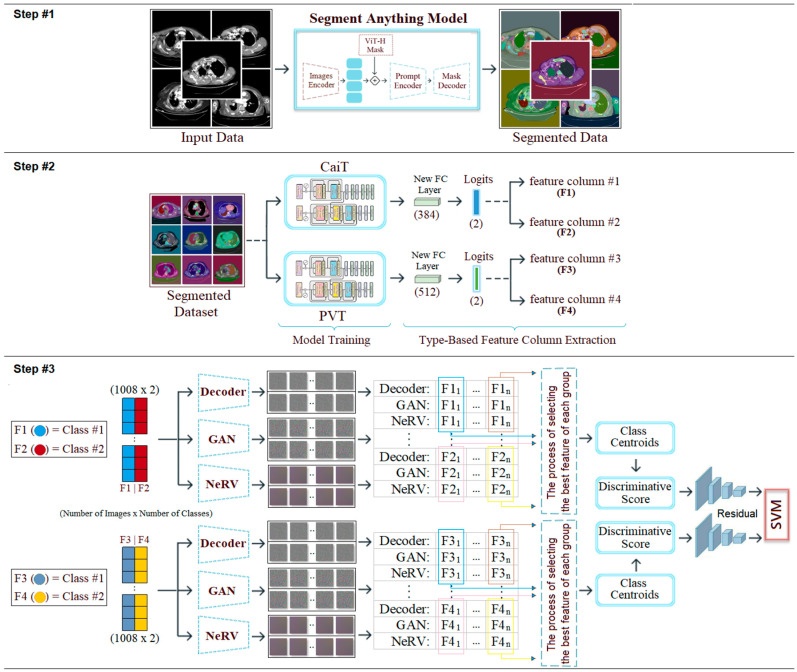
Pipeline steps and general design of the proposed hybrid model.

In the third step (step #3), the F1, F2, F3, and F4 feature columns are processed using deep generative approaches such as Decoder, GAN, and NeRV techniques, and a 2D image is obtained for each feature value. For example, the $“{F1}_{1}$” probability value in the F1 column for the first sample in the dataset is represented by three different images based on Decoder, GAN, and NeRV. Similarly, the $“{F1}_{n}$” value in the F1 column for the last sample in the dataset is also visualized using the same three methods (for this study, n = 1008, which is the total number of data). This process is carried out similarly for the F2, F3, and F4 columns. Since the CaiT model is represented by columns F1 and F2, the best representation is determined among the three images (Decoder, GAN, NeRV) that visually represent each possibility in column F1. For this purpose, the “discriminative score” method is used, and the best representative image is selected for each sample using this method. For example, for the first feature in the F1 column (class #1: mesothelioma) of the CaiT model, the image that provides the best representation according to the discriminative score method may be Decoder-based; the other two images (GAN and NeRV-based) are eliminated. This process is repeated for all samples and applied separately for each model (CaiT, PVT-v2). Subsequently, the best image set representing the F1 and F2 columns obtained with the CaiT model is classified using the residual-based SVM method. Similarly, the image set representing the F3 and F4 columns obtained with the PVT-v2 model is also classified using the same method. The classification performances obtained from the two models are compared, and the overall success of the proposed model is accepted. The general design of the proposed model, showing the pipeline, is presented in Figure 3. The proposed model aims to successfully diagnose mesothelioma by combining innovative approaches in a hybrid manner.

## 3. Experimental Analysis

### 3.1. Experimental Settings

Experimental analyses were performed using the Python 3.12.3 programming language via the Jupyter Notebook interface. The Google Colab server was used during the analysis process. Some important parameters of the models and methods used in the recommended hybrid approach and their values are presented in Table 2. Default parameter values were used for other approaches not specified. In this study, 70% of the dataset was separated as training data and 30% as test data.

The confusion matrix, which is commonly used in classification processes, was used to validate the analyses performed in this study. The metrics associated with the confusion matrix were calculated using the formulas given in Equations (4)–(8) [27,28,29]. The terms used in these equations include positive (p), negative (n), true (t), and false (f). The abbreviations for the metrics obtained are as follows: accuracy (acc), f-score (f-scr), specificity (sp), sensitivity (se), and precision (pre). The acc metric, in particular, stands out as a frequently preferred criterion in evaluations performed on balanced datasets [30,31,32,33].

(4) $$\mathrm{se}=\frac{tp}{fn+tp}$$

(5) $$\mathrm{sp}=\frac{tn}{fp+tn}$$

(6) $$\mathrm{pre}=\frac{tp}{fp+tp}$$

(7) $$f-\mathrm{scr}=\frac{2\times tp}{2\times tp+fn+fp}$$

(8) $$\mathrm{acc}=\frac{tn+tp}{tn+tp+fn+fp}$$

### 3.2. Experimental Results

**Figure 4:**
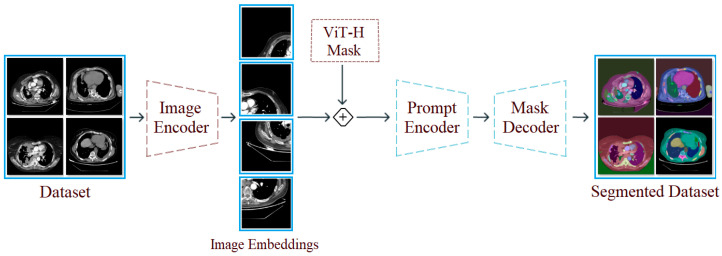
Design showing the general workflow steps of the SAM and its application to a sample dataset.

**Figure 5:**
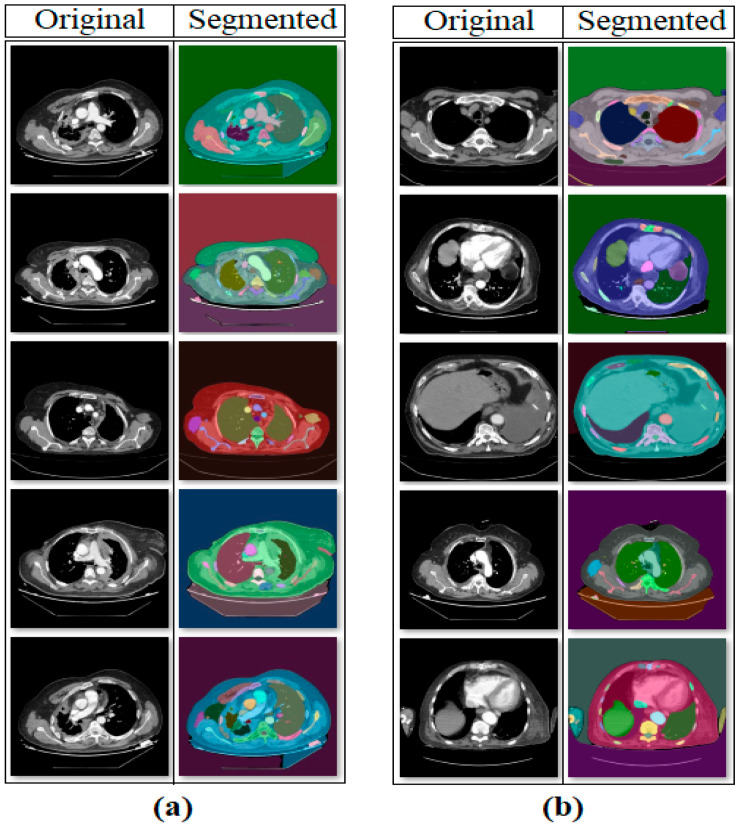
Sample set of segmentation-based data obtained with the SAM: (**a**) mesothelioma, (**b**) non-mesothelioma.

In the first step of the experimental analysis, the original dataset was processed using the SAM approach to create a new segmentation-based dataset. The purpose of this step was to ensure that the model training to be performed in the next step would focus on the regional segments obtained from the CT images and that unnecessary areas would be left in the background by the model. In the CT images where segmentation was applied, a 50% transparency was applied to keep the original regions visible. The internal architecture of the SAM is composed of an Image Encoder, Prompt Encoder, and Mask Decoder. The Image Encoder extracts high-dimensional visual features from the input image, while the Prompt Encoder converts user-specified commands (points, boxes, text) into vectors guiding the segmentation. The Mask Decoder combines these representations to predict segmentation masks for target regions. The processing steps performed using the SAM are shown in Figure 4. Sample images obtained as a result of the segmentation process of the original dataset are shown in Figure 5.

**Figure 6:**
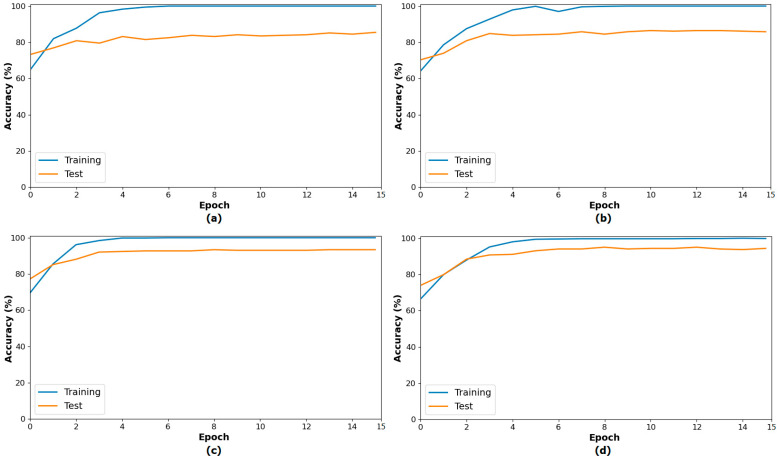
Performance graphs of the CaiT (**a**) and PVT-v2 (**b**) models trained on the original dataset, and the CaiT (**c**) and PVT-v2 (**d**) models trained on the segmented dataset.

**Figure 7:**
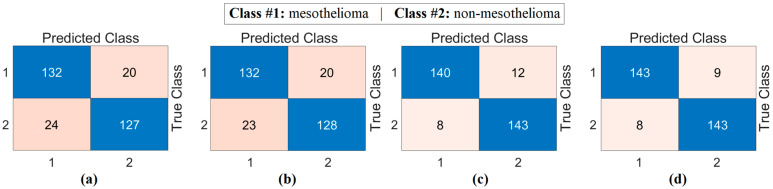
Confusion matrices of the CaiT (**a**) and PVT-v2 (**b**) models trained on the original dataset, and the CaiT (**c**) and PVT-v2 (**d**) models trained on the segmented dataset.

The second step of the experiment involves training transformer-based models (CaiT and PVT-v2) using the original dataset and the segmented dataset. The purpose of this step is to observe whether the segmented dataset contributes to model performance compared to the original dataset. In this regard, both the original and segmented datasets were trained sequentially with the CaiT and PVT-v2 models. The overall accuracy graphs of the models are presented in Figure 6. The confusion matrices obtained by the models as a result of training with the datasets are presented in Figure 7. The macro average metric results obtained from the confusion matrices are presented in Table 3. When examining Figure 6 and Figure 7 and Table 3, it can be observed that the highest success was achieved with the segmented dataset. When the segmented dataset was used, the CaiT model achieved a general accuracy success rate of 93.40%, while the PVT-v2 model achieved 94.39%. Among the transformer models used in the proposed approach, the PVT-v2 model yielded the best results. The CaiT model also demonstrated performance close to that of the PVT-v2 model.

In the final stage of the second step, class-based feature sets were extracted from models trained with segmented datasets (logits layer). The aim at this stage was to extract feature columns equal to the number of classes for each sample, rather than extracting multi-column features from the models. F1 and F2 feature columns were extracted for the CaiT model, and F3 and F4 feature columns were extracted for the PVT-v2 model.

**Figure 8:**
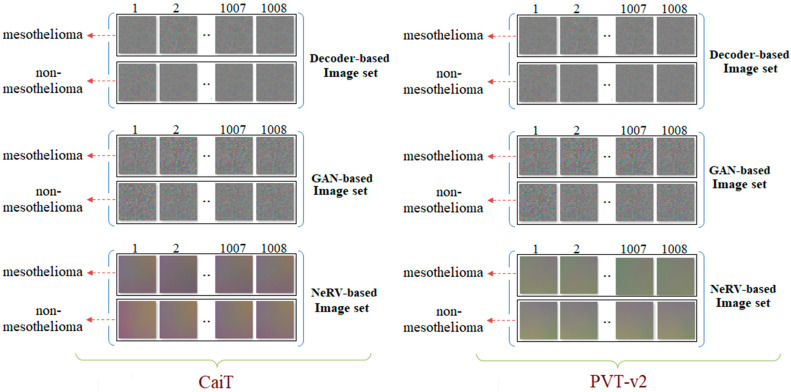
Example images generated from class-based features using deep generative techniques (Decoder, GAN, and NeRV) for the CaiT and PVT-v2 models.

In the third step of the experimental analysis, deep generative techniques (Decoder, GAN, NeRV) were used. Two features representing a sample from the class-based feature sets (F1, F2, F3, F4) obtained from the previous step were converted into images based on Decoder, GAN, and NeRV, respectively. A total of 1008 samples were available, and 1008 decoder, 1008 GAN, and 1008 NeRV-based image sets were obtained for the CaiT model. Similarly, 1008 decoder, 1008 GAN, and 1008 NeRV-based image sets were obtained for the PVT-v2 model. The model-based sample image set is shown in Figure 8.

Figure 8 shows sample images generated from class-based feature sets obtained from the CaiT and PVT-v2 models. These images provide an intermediate visualization of how latent features can be projected onto a 2D image space using deep generative techniques (Decoder, GAN, NeRV). Although these images are not directly used for human interpretation, they serve as a standardized input for the subsequent SVM classification stage. The figure demonstrates that generative methods can preserve the discriminative information in the original features, as evidenced by the high classification accuracies presented in Table 4. Therefore, Figure 8 supports the contribution of the generative transformation step in enhancing the performance of the hybrid model.

**Figure 9:**
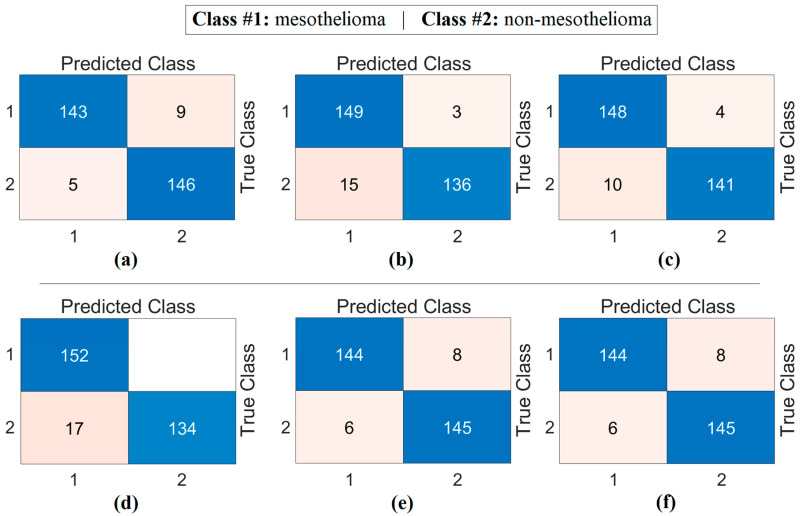
Confusion matrices obtained by classifying images generated from class-based features using deep generative techniques with a residual-based SVM: (**a**) CaiT-Decoder, (**b**) CaiT-GAN, (**c**) CaiT-NeRV, (**d**) PVT-v2-Decoder, (**e**) PVT-v2-GAN, (**f**) PVT-v2-NeRV.

At this stage, image sets created using deep generative techniques from class-based features obtained with both CaiT and PVT-v2 models were classified using a residual-based SVM method. At this stage, since the resolutions of the obtained images were quite low, the deep learning-based ResNet-18-supported SVM method was preferred instead of transformer models (which can make training difficult due to patch separation). The purpose of this process is to determine which imaging technique performed best and to evaluate whether deep generative techniques contributed to classification success. The confusion matrices obtained as a result of the analyses performed at this stage are presented in Figure 9, and the macro average metric results for the relevant matrices are presented in Table 4. Table 4 shows that the results obtained are close to each other and demonstrate high performance. The best results based on CaiT were obtained from both Decoder-based and NeRV-based image sets, with a general accuracy rate of 95.38%. Similarly, the best results based on PVT-v2 were also obtained from both GAN-based and NeRV-based image sets, with a general accuracy rate of 95.38%. In addition, higher accuracy rates were achieved in this stage through analyses performed after the segmentation process. The analyses revealed that the Decoder, GAN, and NeRV techniques contributed positively to the classification process overall.

**Figure 10:**
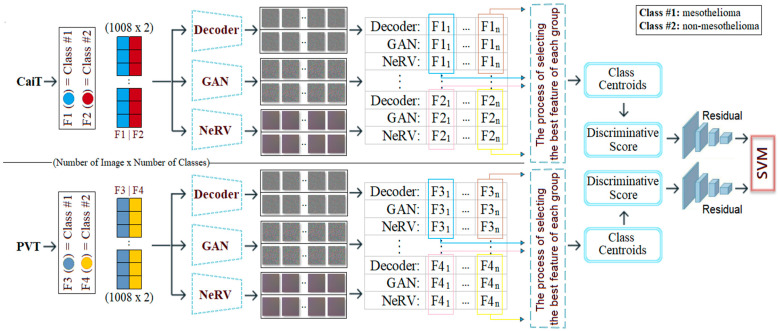
Workflow of the third step: feature visualization, representative image selection, and SVM classification for CaiT and PVT-v2 models.

**Figure 11:**
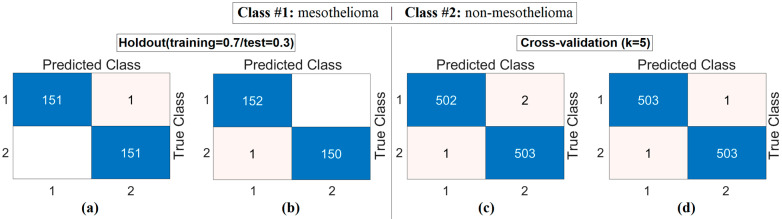
Confusion matrices obtained by classifying images selected from generative image sets using the discriminative score method with the SVM method: (**a**) CaiT-based (holdout), (**b**) PVT-v2-based (holdout), (**c**) CaiT-based (cross-validation), (**d**) PVT-v2-based (cross-validation).

The workflow abstract of the third step performed in the experimental analysis is shown in Figure 10. The class-based feature of each sample was represented using three different visualization methods (Decoder, GAN, and NeRV). Among these visualization types, the image that best represents the relevant sample was determined using the discriminative score method (second stage of the third step). This process was performed separately for both the CaiT model and the PVT-v2 model. The analysis results obtained are presented in Table 5. After determining the best representative image for each sample using the discriminative score method (see Table 5), the classification process was performed using the residual-based SVM method. In the classification process performed using the SVM method, as in previous analyses, 70% of the dataset was allocated for training and 30% for testing. To validate the obtained results and enhance the generalizability of the proposed hybrid approach, the data was further partitioned using the cross-validation method (k = 5) and reclassified using the SVM method. The confusion matrices obtained at this stage are presented in Figure 11, while the relevant metric results are provided in Table 6. When Table 6 is examined, it can be seen that a general accuracy rate of 99.67% was obtained in the best representative image set processed using the holdout technique. On the other hand, a general accuracy rate of 99.80% was achieved in the best representative image set processed using the cross-validation technique (k = 5).

The analyses conducted in the final stage revealed that the discriminative score method works as an effective selection mechanism and thus improves overall performance. The findings confirm the validity of the proposed hybrid model and demonstrate that the model shows high success in the diagnosis of mesothelioma.

**Figure 12:**
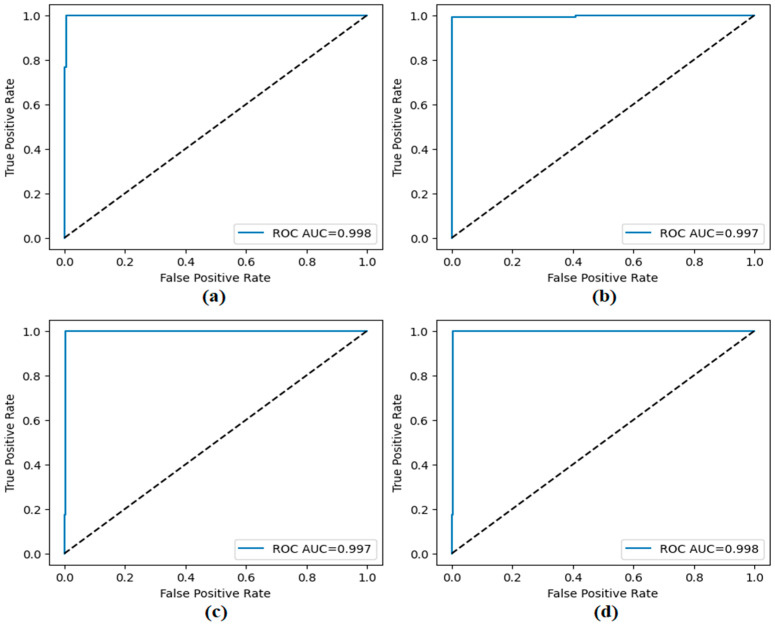
ROC curves obtained by the proposed approach; (**a**) CaiT-based (holdout), (**b**) PVT-v2-based (holdout), (**c**) CaiT-based (cross-validation), (**d**) PVT-v2-based (cross-validation).

**Figure 13:**
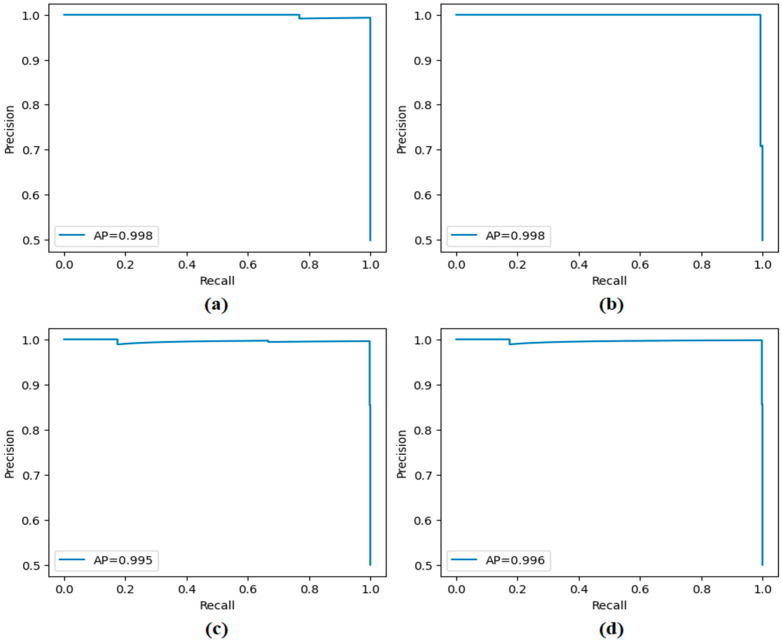
Precision–recall curves obtained by the proposed approach; (**a**) CaiT-based (holdout), (**b**) PVT-v2-based (holdout), (**c**) CaiT-based (cross-validation), (**d**) PVT-v2-based (cross-validation).

**Figure 14:**
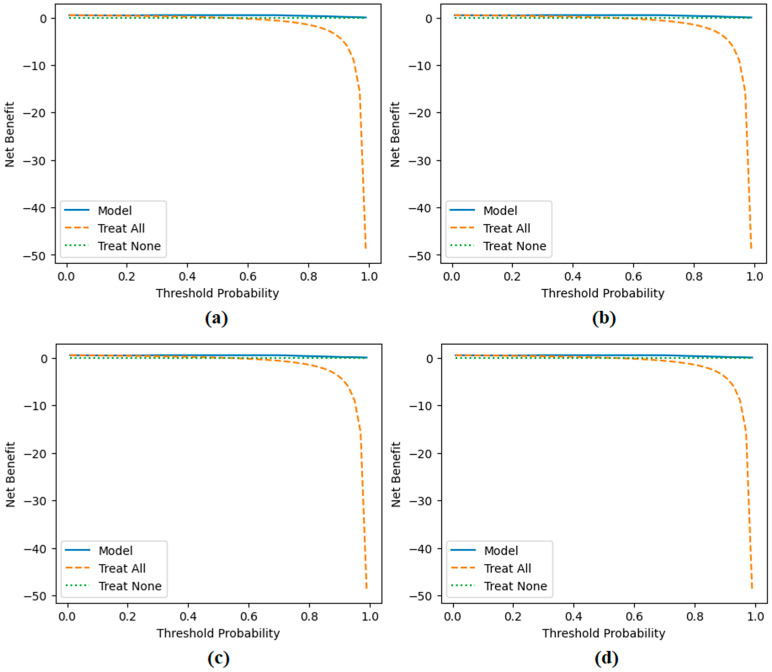
Decision curve analysis results obtained by the proposed approach; (**a**) CaiT-based (holdout), (**b**) PVT-v2-based (holdout), (**c**) CaiT-based (cross-validation), (**d**) PVT-v2-based (cross-validation).

**Figure 15:**
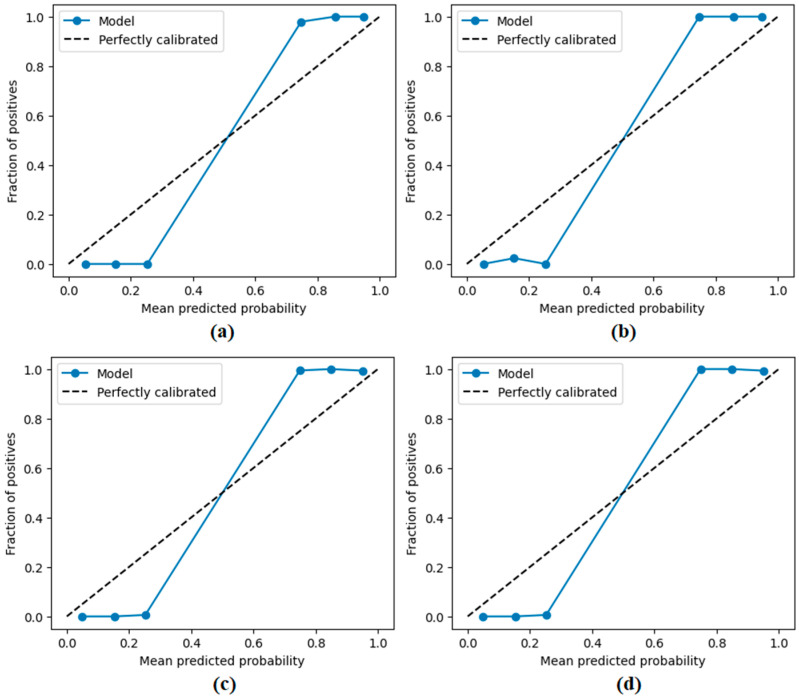
Calibration plots (reliability diagrams) obtained by the proposed approach; (**a**) CaiT-based (holdout), (**b**) PVT-v2-based (holdout), (**c**) CaiT-based (cross-validation), (**d**) PVT-v2-based (cross-validation).

**Figure 16:**
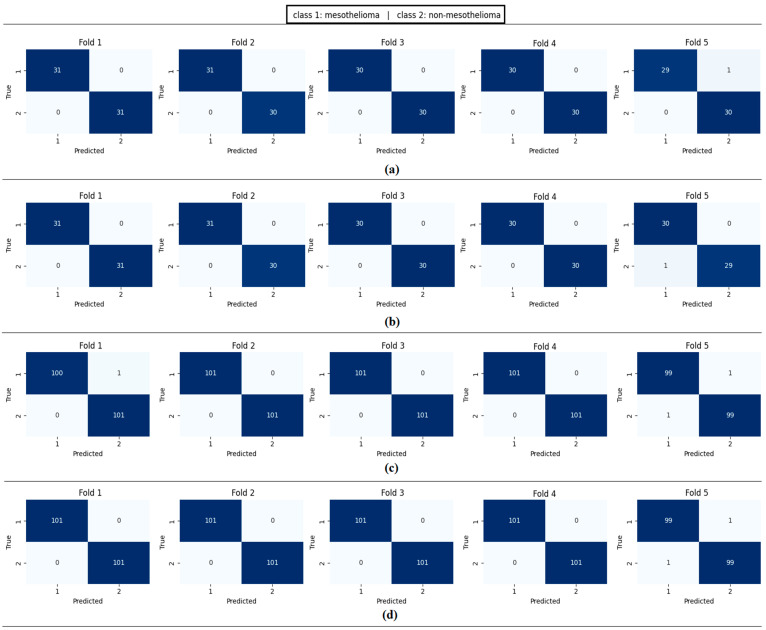
Confusion matrices per fold obtained by the proposed approach; (**a**) CaiT-based (holdout), (**b**) PVT-v2-based (holdout), (**c**) CaiT-based (cross-validation), (**d**) PVT-v2-based (cross-validation).

The performance of the proposed approach was comprehensively evaluated across multiple model and dataset configurations. As reported in Table 7 and Figure 12, Figure 13, Figure 14, Figure 15 and Figure 16, the models consistently achieved excellent results, with AUC values ranging from 0.997 to 0.998, accuracy between 0.997 and 0.998, and both sensitivity and precision exceeding 0.994 across all scenarios. Specificity values were similarly high, ranging from 0.993 to 1.000. Bootstrap-based 95% confidence intervals were narrow for all metrics, further supporting the statistical reliability and robustness of the method.

Figure 11 presents the overall confusion matrices, while Figure 16 provides fold-wise confusion matrices, demonstrating stability and consistency across cross-validation folds. Complementary evaluation curves—including ROC (Figure 12), precision–recall (Figure 13), decision curve analysis (Figure 14), and calibration plots (Figure 15)—confirm the high discriminative ability, reliable probability estimation, and practical utility of the proposed models. To elaborate further, precision–recall curves provide a significant advantage in accurately identifying the positive class (mesothelioma) by simultaneously maintaining high sensitivity and high precision. Calibration curves demonstrate that the probability values predicted by the model are consistent with actual observations and provide reliable decision support. Decision curve analysis shows that the model provides higher net benefit compared to the “Treat All” and “Treat None” strategies across all threshold values, thus supporting clinical benefit. Both CaiT-based and PVT-v2-based models, under holdout and cross-validation schemes, maintained near-perfect performance, indicating strong generalizability and reproducibility of the proposed approach.

**Figure 17:**
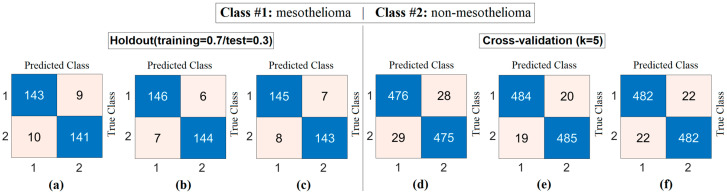
Confusion matrices from ablation analyses on the PVT-v2 model: (**a**) without generative rendering (holdout), (**b**) simpler feature selector (holdout), (**c**) replacing the SVM with a linear classifier (holdout), (**d**) without generative rendering (cross-validation), (**e**) simpler feature selector (cross-validation), (**f**) replacing the SVM with a linear classifier (cross-validation).

To examine the contribution of each component of the proposed approach in greater detail, ablation studies were conducted on the PVT-v2-based architecture that yielded the best results. Figure 17 presents the confusion matrices obtained in scenarios where different components were removed, while Table 8 summarizes the accuracy rates for these scenarios. Removing the generative rendering step and directly feeding the logit values into the SVM resulted in a significant drop in accuracy rates (93.73% holdout and 95.05% cross-validation), highlighting the importance of generative representation learning in extracting discriminative features. Replacing DS-based feature selection with a simpler variance thresholding method yielded higher accuracy compared to removing the generative rendering step; however, it fell short of the results obtained with all model components (95.71% holdout and 96.13% cross-validation). This supports the success of the DS method in identifying the most meaningful features. Furthermore, when logistic regression, a linear method, was used instead of the SVM classifier, a decrease in performance was observed (95.05% retention), indicating that a residual-based SVM is critical for robust classification. The obtained data generally confirms that the combination of generative rendering, discriminative feature selection, and a residual SVM enables the proposed hybrid structure to achieve high performance, reaching 99.80% in cross-validation.

## 4. Discussion

The experimental results demonstrate that the hybrid AI model developed in this study offers an effective approach to overcoming the current challenges in mesothelioma diagnosis. The system’s subcomponents were examined individually to evaluate their impact on diagnostic performance. The results of the study highlight the superiority of AI-based modern techniques in addressing the shortcomings of traditional diagnostic methods. Processes such as image segmentation, deep learning-based feature extraction, and data generation using generative techniques have emerged as critical factors significantly enhancing the model’s diagnostic success. The balanced nature of the dataset ensured that the model’s learning process was conducted in a more robust manner. In the analysis conducted on the proposed hybrid model, a cross-validation technique was applied to the dataset, resulting in an overall accuracy of 99.80%. Similarly, when the holdout technique was applied, an overall accuracy of 99.67% was achieved. The contribution of each stage to the proposed hybrid model was verified by experimental analysis. The contributions and limitations of the proposed approach are as follows:

- Meaningful regions were highlighted in CT images using the SAM method; deep learning models were enabled to focus on these regions and leave meaningless regions in the background.
- Instead of extracting a large number of feature columns from FC layers using recently popular transformer architectures (CaiT, PVT-v2), a new final layer was added that produces feature columns as efficient as the number of classes. This eliminates unnecessary feature columns from the hybrid model.
- Numerical values in feature columns are represented as 2D images using generative techniques such as Decoder, GAN, and NeRV. This method allows approaches that operate on images to extract more diverse and rich numerical content from their architectural structures.
- Alternative image sets were created using generative techniques (Decoder, GAN, and NeRV). The best set was selected using a discriminative scoring method to prevent the model from considering unnecessary images.
- Maintenance and update difficulty: The model’s structure includes many components, which may make it difficult to maintain and develop in the long term.
- The complexity of the decision-making process: Hybrid systems consisting of multiple layers and components can make interpreting the model’s outputs difficult. Such complex structures may also result in additional time costs during the training and inference processes.
- Computational resource requirements: Integrating different architectures, such as the SAM, transformer, GAN, Decoder, and NeRV, may require high processing power and memory in real-time applications.
- The proposed hybrid model is limited by the use of a single-center CT dataset that is not publicly accessible due to ethical constraints. The absence of external validation on independent datasets may limit the generalizability of the findings. Future studies should aim to evaluate the performance of the model on multicenter or publicly available datasets of mesothelioma or other related diseases to validate its broader applicability.

In terms of computational efficiency, applying the most computationally intensive process, SAM-based segmentation, only once during preprocessing provides a significant advantage. The subsequent model training and inference processes were completed within reasonable timeframes using standard GPU hardware. The classification stage provided response times fast enough for clinical applications. Training transformer-based models took approximately two and a half hours in total. Despite their complex hybrid architecture, these times demonstrate the feasibility of the system with commonly available GPU resources. Further optimization for real-time use is planned for future work.

Table 9 summarizes similar studies that recently conducted analyses using CT-based datasets for mesothelioma diagnosis. The datasets used in these studies were obtained with ethical approval and are not publicly available. Studies on CT-based mesothelioma disease diagnosis are limited in the literature.

Kitajima et al. [9] used both PET- and CT-based datasets to diagnose mesothelioma. Although the 3D-CNN model they designed achieved limited success, they could have improved their results by integrating approaches such as preprocessing, feature extraction, and feature selection into their proposed approach. Ye Li et al. [34] developed a diagnostic model to distinguish between MPM and metastatic pleural disease (MPD). In their study, they applied a multivariate logistic regression analysis using 397 CT images and specific clinical features derived from these images, achieving an overall accuracy rate of 95.5%. However, the limited number of CT images used and the lack of model diversity restricted the generalizability of the model’s success and limited performance improvements. The proposed hybrid model offers an architectural structure that integrates both advanced AI techniques and new-generation analysis approaches in CT imaging data. Thanks to this integrated design, our model has demonstrated higher accuracy, segmentation support, and robust classification performance compared to a limited number of similar studies in the literature. The results obtained demonstrate that the proposed model contributes to the clinical decision-making process for mesothelioma.

Although the proposed hybrid model demonstrates high accuracy rates and strong performance, certain limitations associated with the dataset used must be considered. This study was conducted using a single-center CT dataset obtained from Fırat University Research Hospital; in accordance with the ethics committee decision, this dataset cannot be made publicly available. Furthermore, external validation could not be performed due to the lack of an accessible dataset related to CT-based mesothelioma. This situation limits the evaluation of the model’s performance on different data sources. Nevertheless, to maintain the transparency of the study, the methods followed, patient group information, and analysis processes are presented in detail. In future research, it is planned to evaluate the model using multicenter datasets or publicly available CT data for similar diseases to test its validity across different patient groups.

Despite incorporating various sophisticated AI modules, the system’s analyses were conducted using a conventional GPU setup, with inference times kept within a range suitable for clinical applications. Thanks to its modular design, the system allows for future enhancements through the use of lightweight models and compression strategies, potentially lowering computational requirements and supporting real-time use in hospital environments.

Also, the hybrid encoder–decoder architecture we propose, which utilizes class-based feature sets and deep generative techniques, offers a promising approach for diagnosing COVID-19 variants from lung CT images. The study conducted by Fekri-Ershad and Dehkordi proposes a multi-channel deep network that combines tissue and spatial information to detect new COVID-19 variants. In this approach, tissue-based and spatial data are processed in separate channels, which increases diagnostic accuracy [35]. Integrating similar multi-channel architectures into our current framework could further enrich feature representations and improve classification performance. Future work could aim to capture fine details in CT images more effectively by integrating advanced tissue analysis methods, such as local binary patterns (LBPs), into our encoder–decoder architecture. Furthermore, adapting our system to process multiple imaging modalities such as CT and X-ray could make the model more robust and generalizable across different modalities. Such improvements could contribute to the development of more effective diagnostic tools for the early and reliable diagnosis of COVID-19 variants.

## 5. Conclusions

This study emphasizes the current challenges in diagnosing and treating mesothelioma, and shows that a multidisciplinary approach can contribute to an earlier diagnosis. Traditional diagnostic methods have limitations that make it difficult to detect the disease in its early stages, which can delay effective treatment. The proposed hybrid model addresses this issue by integrating new-generation, AI-based approaches, offering a more effective solution. The model aims to enable more accurate and precise mesothelioma diagnoses, particularly through the use of CT images. The hybrid model consists of the following stages: meaningful CT segmentation; training with transformer-based models; class-based feature extraction from models; conversion of each feature into an image using deep generative techniques (Decoder, GAN, and NeRV); and determination of the image that best represents each data sample using a discriminative score method. As a result of these steps, the hybrid model recommended for mesothelioma diagnosis achieved an overall accuracy rate of 99.80%.

The model proposed in this paper provides an innovative contribution to the literature by hybridizing approaches such as meaningful image segmentation, feature extraction using class-based transformer models, and representative image generation using generative AI techniques. The results obtained with the model’s high accuracy rates are at a level that can provide significant support to physicians in clinical decision-making processes. The model’s detailed analysis capability is particularly noteworthy in image-based diagnosis processes. The model’s prominent features are as follows:

- Increased interpretability through visualization of numerical data.
- Production of stable and transparent results thanks to a simplified feature selection mechanism.
- Flexible architecture that can adapt to different clinical scenarios and treatment protocols.
- Elimination of negativity in the decision-making processes of expert physicians; objective decision-making mechanism.
- Prevention of confusion with different disease types in the early diagnosis process.
- These features demonstrate that the model has a broad application potential not only in mesothelioma diagnosis but also in other medical imaging fields.

In future studies, the hybrid model developed in this study will be tested on different types of cancer and various medical imaging modalities to better evaluate its generalization ability. In addition, improving real-time performance and exploring lightweight architectures or model compression techniques will help reduce computational cost and facilitate practical deployment in clinical environments. Another important step will be integrating the model with a user-friendly interface to enhance accessibility for healthcare professionals. Furthermore, the use of explainable artificial intelligence (XAI) techniques will be prioritized to improve interpretability, enabling clinicians and patients to better understand the model’s outputs. Such developments are expected to significantly enhance the effectiveness, efficiency, and reliability of the model in real-world clinical applications. In addition to mesothelioma, the modular design of the proposed hybrid model suggests its potential applicability to other rare cancers that similarly suffer from limited datasets and challenging imaging characteristics. Extending the model to other cancers will be an important direction in our future work.

## Figures and Tables

**Table 1:** Demographic, clinical, and imaging characteristics of the CT dataset used for mesothelioma and non-mesothelioma patients.

Characteristic	Category/Unit	Patients (N, %)
Gender	Male Female	98 74
Age	Year, mean ± SD	56.8 ± 14.6
Stage	1 2 3 4	15 (8.7%) 42 (24.4%) 68 (39.5%) 47 (27.3%)
Histological type	Epithelioid Biphasic Sarcomatoid Not determined	133 (77.3%) 16 (9.3%) 15 (8.7%) 8 (4.7%)
Asbestos exposure	No Yes	41 (23.8%) 131 (76.2%)
Smoking	No Yes	75 (43.6%) 97 (56.4%)
Pain	No Yes	70 (40.7%) 102 (59.3%)
Weight loss	No Yes	57 (33.1%) 115 (66.9%)

**Table 2:** Model/method parameters and values used in the proposed hybrid approach.

Model (Type)/Method	Parameter	Preference/Value
CaiT and PVT-v2	Loss function	Cross Entropy
	Learning rate	10^−4^
	CaiT model type	cait_s24_224
	PVT-v2 model type	pvt_v2_b3
	Optimization	Adam
	Input size	224 × 224
	Epoch	15
	Mini-batch	32
	Random Seed	42 (fixed)
	Learning rate schedule	Constant (no decay)
	Weight decay	Default value (0.0)
	Early stopping	Not applied
	Preprocessing	RandomResizedCrop (224), RandomHorizontalFlip (), ToTensor (), Normalize (mean = [0.485, 0.456, 0.406], std = [0.229, 0.224, 0.225])
	Training rate: testing rate	0.7:0.3
SVM	Kernel function	Cubic
	Kernel scale	Auto
	Box constraint level	1
	Multiclass method	One-vs-One
SAM	Model type	vit_h
ResNet-18 (used with SVM)	Loss function	Cross Entropy
	Optimization	Adam
	Pre-trained weights	ImageNet
	Fine-tuning	Last FC layers
	Epoch	15
	Mini-batch	32
	Input size	224 × 224

**Table 3:** Macro average metric results from the confusion matrix of the transformer models (%).

Transformer Model	Dataset	Se	Sp	Pre	F-Scr	Acc
CaiT	Original	85.47	85.47	85.50	85.47	85.48
PVT-v2	Original	85.81	85.81	85.82	85.81	85.81
CaiT	Segmented	93.40	93.40	93.43	93.40	93.40
PVT-v2	Segmented	94.39	94.39	94.39	94.39	94.39

**Table 4:** Macro average metric results of confusion matrices obtained by classifying image sets generated using deep generative techniques with a residual-based SVM method.

Model	Image Set	Se	Sp	Pre	F-Scr	Acc
CaiT	Generated using Decoder	95.38	95.38	95.41	95.38	95.38
	Generated using GAN	94.05	94.05	94.35	94.05	94.06
	Generated using NeRV	95.37	95.37	95.46	95.38	95.38
PVT-v2	Generated using Decoder	94.37	94.37	94.97	94.37	94.39
	Generated using GAN	95.38	95.38	95.39	95.38	95.38
	Generated using NeRV	95.38	95.38	95.39	95.38	95.38

**Table 5:** Selection list of images represented by discriminative scores in CaiT and PVT-v2 models.

Selection List of the Image Represented by the Discriminative Score in the CaiT Model.	Selection List of the Image Represented by the Discriminative Score in the PVT-v2 Model.
Index,Selected_Method,Label,Image_Path	Index,Selected_Method,Label,Image_Path
0,decoder,Diseased,…\decoder\Diseased\img_0.png	0,nerv,Diseased,…\nerv\Diseased\img_0.png
1,decoder,Diseased,…\decoder\Diseased\img_1.png	1,nerv,Diseased,…\nerv\Diseased\img_1.png
2,gan,Diseased,…\gan\Diseased\img_2.png	2,nerv,Diseased,…\nerv\Diseased\img_2.png
3,decoder,Diseased,…\decoder\Diseased\img_3.png	3,nerv,Diseased,…\nerv\Diseased\img_3.png
4,gan,Diseased,…\gan\Diseased\img_4.png	4,nerv,Diseased,…\nerv\Diseased\img_4.png
5,decoder,Diseased,…\decoder\No disease\img_5.png	5,nerv,Diseased,...\nerv\No disease\img_5.png
6,gan,Diseased,…\gan\No disease\img_6.png	6,nerv,Diseased,…\nerv\No disease\img_6.png
7,gan,Diseased,…\gan\No disease\img_7.png	7,nerv,Diseased,…\nerv\No disease\img_7.png
8,decoder,Diseased,…\decoder\Diseased\img_8.png	8,nerv,Diseased,…\nerv\Diseased\img_8.png
9,decoder,Diseased,…\decoder\No disease\img_9.png	9,nerv,Diseased,…\nerv\No disease\img_9.png
10,decoder,Diseased,…\decoder\No disease\img_10.png	10,nerv,Diseased,…\nerv\No disease\img_10.png
11,decoder,Diseased,…\decoder\Diseased\img_11.png	11,nerv,Diseased,…\nerv\Diseased\img_11.png
12,decoder,Diseased,…\decoder\No disease\img_12.png	12,nerv,Diseased,…\nerv\No disease\img_12.png
13,decoder,Diseased,…\decoder\No disease\img_13.png	13,nerv,Diseased,…\nerv\No disease\img_13.png
14,decoder,Diseased,…\decoder\No disease\img_14.png	14,nerv,Diseased,…\nerv\No disease\img_14.png
15,decoder,Diseased,…\decoder\Diseased\img_15.png	15,nerv,Diseased,…\nerv\Diseased\img_15.png
16,decoder,Diseased,…\decoder\Diseased\img_16.png	16,nerv,Diseased,…\nerv\Diseased\img_16.png
17,decoder,Diseased,…\decoder\No disease\img_17.png	17,nerv,Diseased,…\nerv\No disease\img_17.png
18,decoder,Diseased,…\decoder\No disease\img_18.png	18,nerv,Diseased,…\nerv\No disease\img_18.png
19,decoder,Diseased,…\decoder\No disease\img_19.png	19,nerv,Diseased,…\nerv\No disease\img_19.png
20,decoder,Diseased,…\decoder\No disease\img_20.png	20,nerv,Diseased,…\nerv\No disease\img_20.png
21,decoder,Diseased,…\decoder\No disease\img_21.png	21,nerv,Diseased,…\nerv\No disease\img_21.png
22,decoder,Diseased,…\decoder\Diseased\img_22.png	22,nerv,Diseased,…\nerv\Diseased\img_22.png
23,decoder,Diseased,…\decoder\No disease\img_23.png	23,nerv,Diseased,…\nerv\No disease\img_23.png
24,decoder,Diseased,…\decoder\No disease\img_24.png	24,nerv,Diseased,…\nerv\No disease\img_24.png
25,decoder,Diseased,…\decoder\Diseased\img_25.png	25,nerv,Diseased,…\nerv\Diseased\img_25.png
…	…
993,nerv,No disease,…\nerv\No disease\img_993.png	993,gan,No disease,…\gan\No disease\img_993.png
994,nerv,No disease,…\nerv\Diseased\img_994.png	994,gan,No disease,…\gan\Diseased\img_994.png
995,nerv,No disease,…\nerv\No disease\img_995.png	995,gan,No disease,…\gan\No disease\img_995.png
996,nerv,No disease,…\nerv\Diseased\img_996.png	996,gan,No disease,…\gan\Diseased\img_996.png
997,nerv,No disease,…\nerv\No disease\img_997.png	997,gan,No disease,…\gan\No disease\img_997.png
998,nerv,No disease,…\nerv\No disease\img_998.png	998,gan,No disease,…\gan\No disease\img_998.png
999,nerv,No disease,…\nerv\No disease\img_999.png	999,gan,No disease,…\gan\No disease\img_999.png
1000,nerv,No disease,…\nerv\Diseased\img_1000.png	1000,gan,No disease,...\gan\Diseased\img_1000.png
1001,nerv,No disease,…\nerv\Diseased\img_1001.png	1001,gan,No disease,…\gan\Diseased\img_1001.png
1002,nerv,No disease,…\nerv\Diseased\img_1002.png	1002,gan,No disease,…\gan\Diseased\img_1002.png
1003,nerv,No disease,…\nerv\No disease\img_1003.png	1003,gan,No disease,…\gan\No disease\img_1003.png
1004,nerv,No disease,…\nerv\No disease\img_1004.png	1004,gan,No disease,…\gan\No disease\img_1004.png
1005,nerv,No disease,…\nerv\Diseased\img_1005.png	1005,gan,No disease,…\gan\Diseased\img_1005.png
1006,nerv,No disease,…\nerv\Diseased\img_1006.png	1006,gan,No disease,…\gan\Diseased\img_1006.png
1007,nerv,No disease,…\nerv\Diseased\img_1007.png	1007,gan,No disease,…\gan\Diseased\img_1007.png

**Table 6:** Macro average metric results of confusion matrices obtained by classifying images selected from productive image sets using the discriminative score method with the SVM method (%).

Method	Dataset Technique	Which Model Image Set Was Used?	Se	Sp	Pre	F-Scr	Acc
Discriminative Score	Holdout (training: 0.7/test: 0.3)	CaiT-based	99.67	99.67	99.67	99.67	99.67
		PVT-v2-based	99.67	99.67	99.67	99.67	99.67
	Cross-validation (k = 5)	CaiT-based	99.70	99.70	99.70	99.70	99.70
		PVT-v2-based	99.80	99.80	99.80	99.80	99.80

**Table 7:** Performance metrics of the proposed approach for different model and dataset configurations, including 95% confidence intervals (CIs).

Model/Dataset Configuration	AUC (95% CI)	Accuracy (95% CI)	Sensitivity (95% CI)	Specificity (95% CI)	Precision (95% CI)
CaiT-based (holdout)	0.998 (0.995–1.000)	0.997 (0.990–1.000)	1.000 (1.000–1.000)	0.993 (0.979–1.000)	0.993 (0.979–1.000)
PVT-v2-based (holdout)	0.997 (0.991–1.000)	0.997 (0.990–1.000)	0.994 (0.978–1.000)	1.000 (1.000–1.000)	1.000 (1.000–1.000)
CaiT-based (cross-validation)	0.997 (0.993–1.000)	0.997 (0.993–1.000)	0.998 (0.994–1.000)	0.996 (0.990–1.000)	0.996 (0.990–1.000)
PVT-v2-based (cross-validation)	0.998 (0.995–1.000)	0.998 (0.995–1.000)	0.998 (0.994–1.000)	0.998 (0.994–1.000)	0.998 (0.994–1.000)

**Table 8:** Ablation analyses on different components of the proposed hybrid approach performed on the PVT-v2 backbone. (The PVT-v2 model was chosen as it achieved the highest performance in the proposed approach.)

Experiment Setup	Dataset Technique	Acc (%)
Proposed Hybrid Model (SAM + generative rendering + DS + SVM)	Holdout (CaiT-based)	99.67
	Holdout (PVT-v2-based)	99.67
	Cross-val. (CaiT-based)	99.70
	Cross-val. (PVT-v2-based)	99.80
Without Generative Rendering (direct logits → SVM)	Holdout	93.73
	Cross-val.	95.05
Simpler Feature Selector (variance threshold instead of DS)	Holdout	95.71
	Cross-val.	96.13
Replacing SVM with Linear Classifier (logistic regression)	Holdout	95.05
	Cross-val.	95.63

**Table 9:** Similar studies in the literature using CT images for the detection of mesothelioma.

Paper	Year	Number of Patients	Number of Classes	Model/Method	Acc (%)
Kitajima et al. [9]	2021	875	2	3D-CNN	77.3
Ye Li et al. [34]	2022	397	2	Multivariate logistic regression	95.5
This study	2025	1008	2	SAM and transformers and class-based feature extraction and deep generative and discriminative score	99.80 (cross-val.)
					99.67 (holdout)

## Data Availability

The original contributions presented in the study are included in the article; further inquiries can be directed to the corresponding authors.
